# Study on the Growth and Enterotoxin Production by *Staphylococcus aureus* in Canned Meat before Retorting

**DOI:** 10.3390/toxins11050291

**Published:** 2019-05-23

**Authors:** Luca Grispoldi, Paul Alexanderu Popescu, Musafiri Karama, Vito Gullo, Giusi Poerio, Elena Borgogni, Paolo Torlai, Giuseppina Chianese, Anna Giovanna Fermani, Paola Sechi, Beniamino Cenci-Goga

**Affiliations:** 1 Medicina Veterinaria, Laboratorio di Ispezione degli Alimenti di Origine Animale, Università degli Studi di Perugia, 06126 Perugia, Italy; vito93.v@gmail.com (V.G.); giusi.poerio@gmail.com (G.P.); borgogni.ele@gmail.com (E.B.); si.to06@libero.it (P.T.); giusychianese@hotmail.com (G.C.); paola_sechi@outlook.it (P.S.); beniamino.cencigoga@unipg.it (B.C.-G.); 2 Faculty of Biotechnology, University of Agronomical Science and Veterinary Medicine, 011464 Bucharest, Romania; paul.alex.popescu@gmail.com; 3 Faculty of Veterinary Science, Department of Paraclinical Sciences, University of Pretoria, Onderstepoort 0110, South Africa; Musafiri.Karama@up.ac.za; 4 Department of prevention, Azienda Unità Sanitaria Locale Latina, 04012 Lazio, Italy; angiferm@gmail.com

**Keywords:** *Staphylococcus aureus*, canned meat, enterotoxin, HACCP

## Abstract

Possible contamination by *Staphylococcus aureus* of the production environment and of the meat of a canned meat production factory was analysed. A total of 108 samples were taken from nine critical control points, 13 of them were positive for *S*. *aureus*. None of the isolates produced enterotoxins. To determine how much time can elapse between can seaming and sterilisation in the autoclave without any risk of enterotoxin production by *S*. *aureus*, the growth and enterotoxin production of three enterotoxin A producing strains of *S. aureus* (one ATCC strain and two field strains) in canned meat before sterilisation was investigated at three different temperatures (37, 20 and 10 °C). Two types of meat were used, one with and one without sodium nitrite. In the canned products, the spiked bacteria spread throughout the meat and reached high levels. Enterotoxin production was shown to start 10 hours after incubation at 37 °C and after 48 h after incubation at 20 °C; the production of enterotoxin was always detected in the transition between the exponential and the stationary growth phase. At 10 °C, the enterotoxin was never detected. The statistical analysis of the data showed that the difference between the two different types of meat was not statistically significant (*p* value > 0.05). Since it is well known that following heat treatment, staphylococcal enterotoxins, although still active (in in vivo assays), can be undetectable (loss of serological recognition) depending on the food matrix and pH, it is quite difficult to foresee the impact of heat treatment on enterotoxin activity. Therefore, although the bacteria are eliminated, the toxins may remain and cause food poisoning. The significance of the results of this study towards implementing good manufacturing practices and hazard analysis critical control points in a canned meat factory are discussed with reference to the management of pre-retorting steps after seaming.

## 1. Introduction

Processed meats are protein-rich foods, which can serve as an excellent culture media for the growth of microorganisms [1]. Contamination by microorganisms can be exacerbated during the canning process, especially if the end product presents low acidity and is maintained in conditions of temperature abuse [2]. Contamination may occur not only during meat processing if manufacturing practices are poor, but also during the processes of transportation, storage and handling. There are several bacterial species, which are known to be able to contaminate canned meat, e.g., *Clostridium* spp., *Listeria* spp., *Bacillus* spp., *Escherichia coli* and *Staphylococcus aureus* [3,4,5].

Among those pathogens, staphylococci are of particular interest, as they are one of the most frequent microbial contaminants isolated from small- and medium-sized meat processing factories around the world [2,6]. *S*. *aureus* represents a serious hazard for the end consumer, as it is able to produce enterotoxins, which are stable at high temperatures (e.g., Crude enterotoxin A remains active at 100 °C for 2 h in broth and at 121 °C for 28 min in mushrooms) and can also resist under many environmental conditions (low pH, freezing, drying), in which *S. aureus* strains do not survive [7,8]. They are also resistant to human proteolytic enzymes and retain their activity in the digestive tract after ingestion [9]. The amount of enterotoxin required to cause the illness in susceptible subjects can be as little as 20–100 ng [10]. A fraction of the strains of *S*. *aureus* are also able to persist in the factory environment by forming biofilm [11].

To date, a total of 23 distinct SEs have been identified, based on their antigenicity (SEA to SElY) [12]. Among these, staphylococcal enterotoxin A (SEA) is the most frequently reported in cases of food poisoning [13,14].

The literature has reported several past cases of food poisoning caused by *S*. *aureus* contaminated canned meat [15,16,17]. In more recent years, despite the strict regulations on the processing and canning of food products, there are still cases of food poisoning related to the consumption of canned foods: e.g., the EU Rapid Alert System for Food and Feed (RASFF) reported the presence of *S. aureus* and staphylococcal enterotoxins in various meat products in Germany (2005) and the Netherlands (2007). 

There is little information available in literature about the growth, survival and enterotoxin production by *S*. *aureus* in canned meat. Most of the available data is based on models consisting of liquid cultures, where the bacteria are in a planktonic state. Recent studies have shown that there are significant differences between those models and bacteria in a food matrix, where the association with surfaces and tissue and the communication with other bacteria by means of molecular signalling are prevalent [18,19].

In this study, we analysed the possible contamination by *S*. *aureus* of the production environment and of the meat in a large canned meat production factory. We investigated the behaviour of three enterotoxigenic *S*. *aureus* strains spiked in canned meat with and without sodium nitrite (a food additive used as a preservative and colour fixative in cured meat) at different temperatures, in order to implement good manufacturing practices and the hazard analysis and critical control point system at the factory, with specific attention to the management of pre-retorting steps after seaming.

## 2. Results

Table 1 shows the results of the microbiological analysis of the meat samples and swabs from the canned meat factory. The mesophilic flora ranged from a maximum of 3.91 ± 1.79 CFU g^−1^ in the meat on the belt after the metal detector to a minimum of 1.51 ± 0.88 CFU (cm^2^)^−1^ in the belt swabs. *Micrococcus* spp. reached a concentration of 2.84 ± 1.72 CFU g^−1^ in the meat on the belt after the metal detector. At the same point, *Staphylococcus* spp. reached a maximum concentration of 2.52 ± 1.62 CFU g^−1^. No bacteria were found in the aspic samples taken from the doser. *S. aureus* isolates came from five out of the nine kinds of samples, for a total of 13 isolates. Six of those isolates came from samples of frozen cooked beef at reception. None of the *S*. *aureus* isolates produced enterotoxins.

The initial concentration of *S. aureus* spiked ranged between 3.94 ± 0.16 CFU g^−1^ and 4.19 ± 0.11 CFU g^−1^ in meat with sodium nitrite and between 3.85 ± 0.27 CFU g^−1^ and 4.26 ± 0.17 CFU g^−1^ in meat without sodium nitrite. 

**Figure 1:**
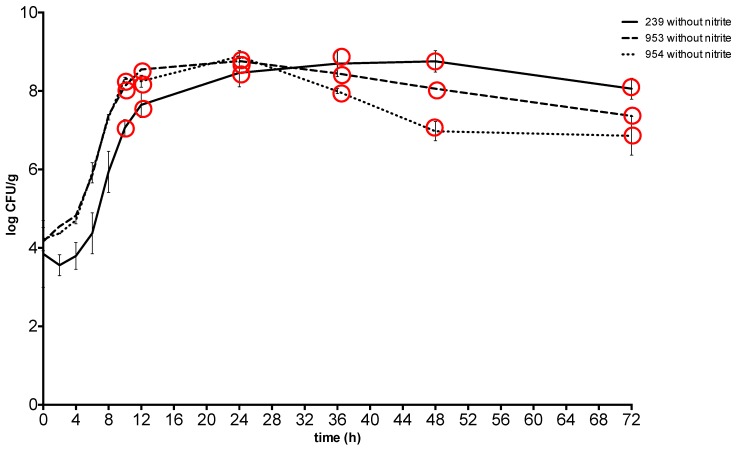
Growth and enterotoxin production of *S*. *aureus* at 37 °C in meat without sodium nitrite. Red circle: enterotoxin production. 239: *S. aureus* ATCC 29213; 953: *S. aureus* field strain; 954: *S. aureus* field strain.

**Figure 2:**
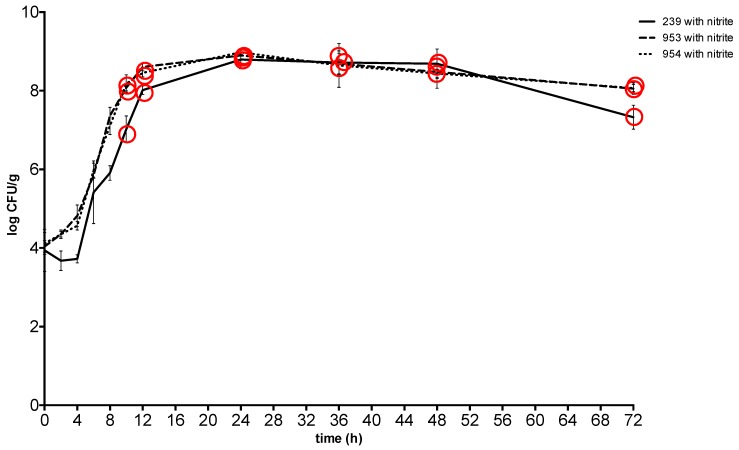
Growth and enterotoxin production of S. aureus at 37 °C in meat with sodium nitrite. Red Circle: enterotoxin production. 239: *S. aureus* ATCC 29213; 953: *S. aureus* field strain; 954: *S. aureus* field strain.

At 37 °C, *S*. *aureus* counts reached the plateau phase after 24 h at a concentration between 8.79 ± 0.08 CFU g^−1^ and 8.98 ± 0.03 CFU g^−1^ in the meat with sodium nitrite and between 8.46 ± 0.14 CFU g^−1^ and 8.88 ± 0.1 CFU g^−1^in the meat without nitrite, and maintained similar values throughout the experiment. The toxin was detected from the 10-hour at a concentration of *S. aureus* between 7.02 ± 0.15 CFU g^−1^ and 8.18 ± 0.11 CFU g^−1^ in the meat with sodium nitrite and at the same time at a concentration between 7.06 ± 0.09 CFU g^−1^ and 8.32 ± 0.02 CFU g^−1^ in the meat without nitrite (Figure 1 and Figure 2). 

**Figure 3:**
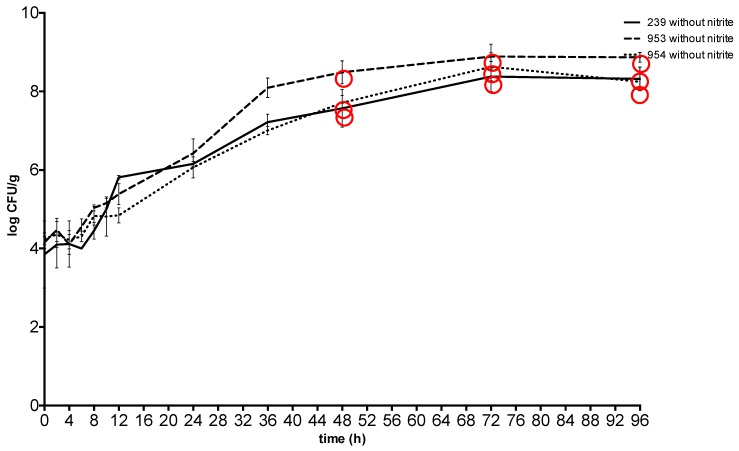
Growth and enterotoxin production of S. aureus at 20 °C in meat without sodium nitrite. Red circle: enterotoxin production. 239: *S. aureus* ATCC 29213; 953: *S. aureus* field strain; 954: *S. aureus* field strain.

**Figure 4:**
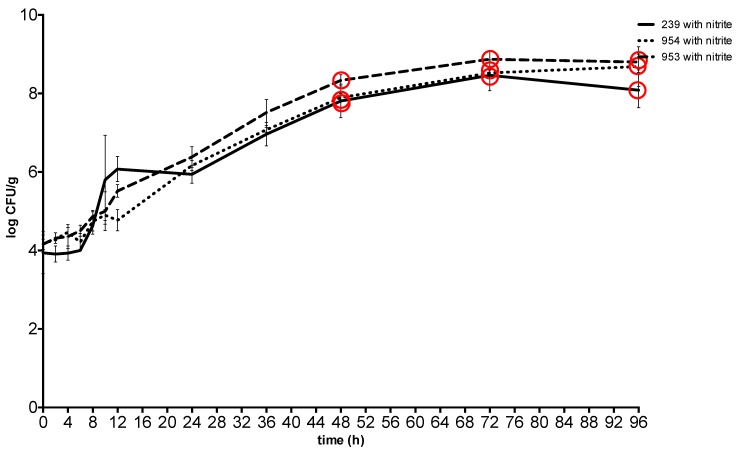
Growth and enterotoxin production of S. aureus at 20 °C in meat with sodium nitrite. Red circle: enterotoxin production. 239: *S. aureus* ATCC 29213; 953: *S. aureus* field strain; 954: *S. aureus* field strain.

At 20 °C, the plateau phase was reached 72 h after spiking at a concentration between 8.47 ± 0.16 CFU g^−1^ and 8.87 ± 0.11 CFU g^−1^in meat with sodium nitrite and at the same time at a concentration between 8.38 ± 0.16 CFU g^−1^ and 8.88 ± 0.16 CFU g^−1^ in meat without sodium nitrite. The toxin was detected from the 48th hour with a *S*. *aureus* concentration between 7.81 ± 0.19 CFU g^−1^ and 8.34 ± 0.07 CFU g^−1^ in the meat with sodium nitrite and between 7.57 ± 0.19 CFU g^−1^ and 8.49 ± 0.15 CFU g^−1^ in the meat without nitrite (Figure 3 and Figure 4).

**Figure 5:**
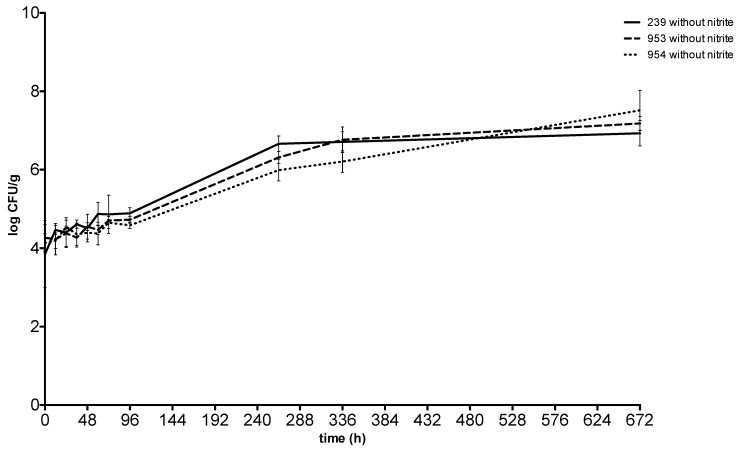
Growth and enterotoxin production of S. aureus at 10 °C in meat without sodium nitrite. 239: *S. aureus* ATCC 29213; 953: *S. aureus* field strain; 954: *S. aureus* field strain.

**Figure 6:**
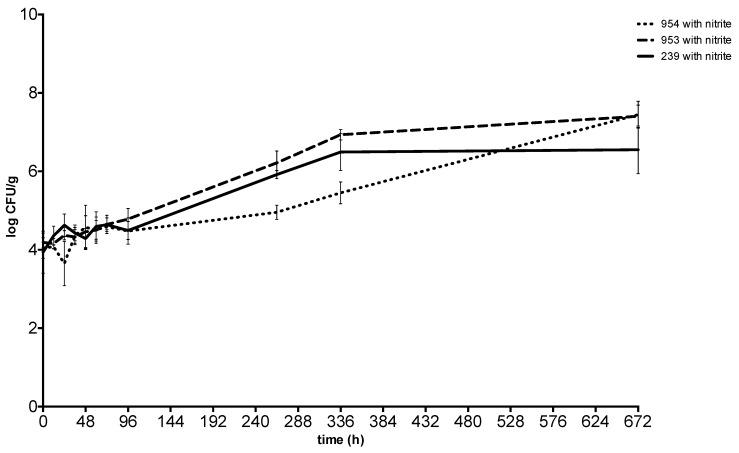
Growth and enterotoxin production of S. aureus at 10 °C in meat with sodium nitrite. 239: *S. aureus* ATCC 29213; 953: *S. aureus* field strain; 954: *S. aureus* field strain.

After 28 days at 10 °C, the concentration of *S. aureus* was between 6.55 ± 0.27 CFU g^−1^ and 7.44 ± 0.14 CFU g^−1^ in the meat with sodium nitrite and between 6.93 ± 0.19 CFU g^−1^ and 7.52 ± 0.21 CFU g^−1^ in the meat without sodium nitrite. Enterotoxin was never detected throughout the incubation at 10 °C (Figure 5 and Figure 6).

The statistical analysis of the data showed that the difference in the bacterial growth rate between the meat with sodium nitrite and the meat without sodium nitrite was not statistically significant, with a constant *p* value of >0.05.

## 3. Discussion

The data on the *S*. *aureus* contamination of the meat and the production environment is consistent with other studies: Koreňová et al. (2015) [6] reported 5 out of 144 samples positive for *S. aureus* in a small meat processing factory. 

The growth profile and enterotoxin production of the three *S*. *aureus* strains tested in canned meat is similar to what is reported by other studies. Mansfield et al. (1983) [16] tested the growth and survival of *S*. *aureus* in canned meat at 15, 22, 30 and 37 °C and reported that the colony counts increased to a maximum of about 10^8^ CFU g^−1^ at all temperatures, and the maximum concentration was reached first at the higher temperature. Enterotoxin production was detected when the colony count reached a concentration of >10^6^ CFU g^−1^. Wallin-Carlquist et al. (2010) [1] investigated the growth of *S*. *aureus* in different meat products stored at room temperature: starting from an initial concentration of approximately 10^4^ CFU g^−1^, the bacteria reached a concentration of 8.87 log CFU cm^−2^ in boiled ham and 8.52 log CFU cm^−2^ in smoked ham. The presence of SEA was detected after 24 h and for the entire incubation period. In our study, the bacteria reached similar concentrations after the same period of time and the production of enterotoxin was always detected in the transition between the exponential and the stationary growth phase.

Comparing our results to data based on a model consisting of liquid cultures, some differences can be observed. Tsutsuura et al. (2013) [20] incubated eleven SEA producer strains of *S*. *aureus* in BHI broth in temperatures ranging from 10 to 37 °C. SEA produced by these eleven strains were detected after 3 weeks of incubation at 10 °C at a concentration of 7.85 log CFU mL^−1^, after 3–8 days at 15 °C at a concentration of 9.34 log CFU/mL^−1^, after 30–58 h at 20 °C at a concentration of 6.96 log CFU/mL^−1^, and after 6–8 h at 37 °C at a concentration of 7.42 log CFU mL^−1^ with an inoculum size of 10^2^ CFU mL^−1^. In our study, the growth and enterotoxin production of *S. aureus* in canned meat was slower at 37 °C and 20 °C, whereas the enterotoxin was never detected at 10 °C. 

Canned meat has different characteristics to a broth, e.g., nutrient availability, pH, salt content and water activity [21]: furthermore, whereas the bacteria in the broth are in a planktonic state, in the meat they grow in multi-cellular communities and can form biofilm. The enterotoxin A gene (*sea*) is carried by a family of temperate bacteriophages [22]. The bacteriophage is inserted in the bacterial chromosome as a prophage. Many studies have demonstrated that a stressful condition found in the food matrix can influence SEA production [23]. Tsutsuura et al. (2013) [20] demonstrated that SEA production by *S*. *aureus* is influenced not only by the intrinsic property of the strain, but also by the incubation temperature and inoculum size.

The statistical analysis of the data did not show any differences between the tests in meat with and without sodium nitrite. These results are consistent with previous studies, which demonstrated that there is no evidence of the inhibition of *S*. *aureus* growth in canned meat products by similar concentrations of nitrites [24].

## 4. Conclusions

The strict regulations on processing and canning of food products and the improvement of the HACCP system and of good manufacturing practices have strongly increased food safety. However, contamination of the meat before retorting by *S*. *aureus* is still possible and may represent a risk for the end consumer, due to its ability to produce highly thermostable toxins, which may not be inactivated during can sterilisation. Our study demonstrated that a SEA producer strain of *S*. *aureus* takes at least 10 h to produce detectable quantities of toxin at 37 °C and 48 h at 20 °C under the conditions tested, leaving quite a wide range of time to manage pre-retorting steps after seaming. Considering the fact that our study never detected SEA at 10 °C, even though the colony count reached a high level, it has demonstrated once again that the behaviour of *S*. *aureus* often differs in a complex food matrix than in liquid culture broth.

## 5. Materials and Methods

### 5.1. Microbiological Analysis of Samples from the Factory

In this study, the production flow chart of a canned meat production factory was analysed. Nine critical points for the contamination of meat by *S. aureus* (frozen cooked beef at reception, sliced beef at the slicer, belt, piston, frozen cooked beef at thawing, defrost water, cooked beef after thawing, meat on the belt after metal detector, aspic at the doser) were identified. A total of 108 samples were taken from these points, in order to determine the microbial flora and the possible presence of *S*. *aureus*. The samples were sent to the laboratory in a refrigerated container. To detect the quantitative and qualitative presence of the bacteria, swabs and food products were homogenised in a stomacher in 90 mL of peptone water (PW, Oxoid, Basingstoke, Hampshire, UK). 10-fold dilutions were made using sterile tubes with 9 mL of Maximum Recovery Diluent (MRD, Oxoid, Basingstoke, UK). Dilutions were inoculated in triplicate on Plate Count Agar (PCA, Oxoid) and Baird Parker Agar (BP, Oxoid), prepared with the addition of egg yolk tellurite emulsion (Liofilchem, Roseto degli Abruzzi, TE, Italy) using the spread plate technique, and incubated at 37 °C for 48 h. The colonies were then counted on all the plates, using a colony count viewer (Petri light, PBI, Milan, Italy) and colony counter pen (Colony Count, PBI, Milan, Italy). A shiny, greyish-black, convex colony, measuring from 1–1.5 mm up to 3 mm in diameter with a narrow, white, unbroken margin, surrounded by a 2–5 mm clear area, was identified as suspected *S. aureus* and confirmed by complementary biochemical tests with API 20 STAPH (BioMéhrieux, Marcy-l’Etoile, France).

### 5.2. Canned Meat Spiking

To determine how much time can elapse between can seaming and sterilisation in the autoclave without any risk of enterotoxin production by *S*. *aureus* and with the consequent risk of food poisoning for the consumer, the ATCC 29213 strain of *Staphylococcus aureus* (internal reference #239) and two field strains (internal reference #953 and #954)*,* all producers of staphylococcal enterotoxin A (SEA), belonging to the collection of the Food Inspection laboratory of the Department of Veterinary Medicine, University of Perugia, were used to spike samples of the canned meat used throughout the experiments. 

Two different types of canned meat, one with and one without sodium nitrite (20 ppm), were used to study the growth rate and enterotoxin production of *Staphylococcus aureus* at 10 °C, 20 °C and 37 °C to simulate the actua l temperatures that can be reached after aspic addition, seaming and during the layover before sterilisation. The canned meat samples presented a pH value of 5.83 and an a_w_ value of 0.971. Six replications were made for each trial (with and without sodium nitrite) and for each temperature on six different days. The strains were thawed and cultured in Brain Heart Infusion Broth (BHI, Oxoid). After a 48-h incubation at 37 °C, *S. aureus* reached a concentration of 10^8^–10^9^ CFU mL^−1^. Ten-fold dilutions were made to obtain the correct concentration for spiking. The cans were opened in a sterile environment and 270 g of each canned meat sample (with and without sodium nitrite) were weighed in a sterile bag. Then, the meat samples were spiked with *S. aureus* to achieve a final concentration of 10^3^–10^4^ CFU mL^−1^ in the meat. The bags with the mixture of meat and *S. aureus* solution were homogenised for 1 min at 260 rpm in the Stomacher 400 (PBI International, Milan). 20 g of the sample were then placed in sterile glass jars (12 per sample). The jars were individually placed in vacuum bags in order to recreate can conditions and incubated at 37 °C for the first trial, at 20 °C for the second and at 10 °C for the third. Control samples with only canned meat were taken for each type of meat and incubated at the same temperature as the inoculated samples. The times of analysis for each trial were taken at 0, 2, 4, 6, 8, 10, 12, 24, 36, 48 and 72 h, plus 96 h for incubation at 20 °C and on day-11, day-14 and day-28 for incubation at 10 °C. 

At each time of analysis, two different tests were conducted on the samples: the detection of the production of enterotoxin and a microbiological analysis to determine the growth of *S. aureus*.

### 5.3. Study of the Growth Rate

For the growth rate study, a 10 g portion of inoculated meat was transferred from each sample into sterile tubes containing 90 mL of Maximum Recovery Diluent (MRD, Oxoid) and 10-fold dilutions were prepared. Dilutions were inoculated in triplicate on Baird Parker Agar (BP, Oxoid) using the spread plate technique and incubated at 37 °C for 48 h. Colonies were then counted on all the plates, using a colony count viewer (Petri light, PBI, Milan) and colony counter pen (Colony Count, PBI, Milan). All values were converted into logs and the arithmetic mean was calculated for each sampling. All values were analysed with a Graph Pad In Stat, version 3.0b for Mac OS X (GraphPad Software, San Diego, CA, USA); the graphs and the statistical analysis (paired samples *t*-tests) were obtained with a Graph Pad Prism, version 6.0d for Mac OS X (GraphPad Software). 

### 5.4. Enterotoxin Detection

For the study of the enterotoxin production, 10 g of each sample were weighed and then homogenised with 15 mL of PBS buffer. The prepared samples were shaken for 15 min and then centrifuged for 5 min at 3500 rpm at 10 °C. The supernatant was then collected and immediately analysed. A RIDASCREEN SET Total (R-Biopharm, Melegnano, Milan, IT), an enzyme immunoassay for the combined detection of *S. aureus* enterotoxins not only in fluid and solid foods, but also in bacterial cultures, was used to determine the production of enterotoxin. A photometrical interpretation of the results was made by measuring the absorbance at 450/630 ± 10 nm with a microwell plate photometer (SEAC, Radim Group, Freiburg, Germany). The cut-off value to evaluate the results as negative or positive of 0.17 was calculated by adding 0.15 to the value of the negative control (0.020). A sample was considered positive when the test was valid and the absorbance of the sample was higher than, or equal to, the cut-off value.

## Figures and Tables

**Table 1:** Results of the microbiological analysis.

Sampling Points	Number of Samples	Mesophilic (Mean ± SD)	*Staphylococcus* spp. (Mean ± SD)	*Micrococcus* spp. (Mean ± SD)	*Staphylococcus aureus* (Number of Isolated Strains)
Frozen cooked beef at reception	15	2.94 ± 1.57	1.92 ± 1.39	1.94 ± 1.76	6
Sliced beef at the slicer	12	2.06 ± 1.45	0.47 ± 0.85	1.24 ± 1.36	0
Belt swabs	9	1.51 ± 0.88	0.49 ± 0.78	0.34 ± 0.68	1
Piston swabs	9	1.52 ± 1.24	0.26 ± 0.51	0.19 ± 0.57	0
Frozen cooked beef at thawing	36	2.86 ± 0.83	1.49 ± 1.39	0.63 ± 1.12	4
Defrost water	6	2.02 ± 1.47	0.91 ± 0.74	0.00 ± 0.00	1
Cooked beef after thawing	6	3.12 ± 0.39	0.41 ± 1.01	0.33 ± 0.82	0
Meat on the belt after metal detector	12	3.91 ± 1.79	2.52 ± 1.62	2.84 ± 1.72	1
Aspic at the doser	3	0.00 ± 0.00	0.00 ± 0.00	0.00 ± 0.00	0
